# Physicochemical and Textural Enhancement of Whole Wheat Bread Using Date Palm Gum: A Study on a Novel Natural Hydrocolloid

**DOI:** 10.3390/foods14223968

**Published:** 2025-11-19

**Authors:** Durga Jumble, Sampurna Ghosh, Ahana Paul, Debmalya Banerjee, Anish Kumar, Bala Chakravarthy Neelapu, Sivaraman Jayaraman, Maciej Jarzębski, Krishan Kumar, Kunal Pal

**Affiliations:** 1 Department of Life Sciences, Parul Institute of Applied Science, Parul University, Vadodara 391760, Gujarat, India; durgajumbledj@gmail.com (D.J.); ahanapaul170801@gmail.com (A.P.); 2 Department of Biotechnology and Medical Engineering, National Institute of Technology Rourkela, Rourkela 769008, Odisha, India; ghoshsampurna529@gmail.com (S.G.); debmalyab60@gmail.com (D.B.); anishkr6201@gmail.com (A.K.); bala3605@gmail.com (B.C.N.); mountshiva@gmail.com (S.J.); 3 Department of Physics and Biophysics, Faculty of Food Science and Nutrition, Poznań University of Life Sciences, Wojska Polskiego 38/42, 60-637 Poznań, Poland; maciej.jarzebski@up.poznan.pl

**Keywords:** whole wheat bread, date tree gum, hydrocolloids, phenolic oxidation, starch–protein complexes

## Abstract

This study explores the effect of date palm gum (DPG) as a novel functional ingredient for whole wheat bread (WWB) to enhance its physicochemical and textural properties. Herein, samples containing varying concentrations of DPG (0–3% *w*/*w*) were prepared and analyzed, out of which D2 (containing 1% *w*/*w* DPG) exhibited superior qualities. Microscopic studies showed that D2 exhibited improved crumb aeration, suggesting better fermentation than the others. Moisture analysis revealed that D2 retained a higher quantum of moisture (50.06 ± 0.41%). Further, the colorimetric study showed that increasing DPG concentration led to a corresponding decrease in L* values (46.69 ± 0.13) due to the combined effect of Maillard browning and the inherent color of DPG. Analysis of FTIR spectra confirmed stable interactions of DPG and starch–protein complexes in D2. Stress relaxation exhibited that D2 had the highest initial (F_0_; 162.95 ± 1.70 g) and residual (F_60_; 95.81 ± 3.94 g) forces, indicating that it maintained its structure under stress. In gist, DPG exhibited strong potential as a natural hydrocolloid that could be explored to develop functional bakery products.

## 1. Introduction

With growing global awareness of health and nutrition, enhancing the nutritional profile of staple foods such as bread has become a key focus in the functional food sector. Bread is a universally consumed staple with significant and growing global market value, driven by consumer preferences for healthier, nutrient-rich options. This growth is expected to be driven by population increase and shifting consumer preferences towards healthier and nutrient-rich food options. In this regard, whole wheat flour (WWF) is increasingly preferred over refined flour, as it retains the nutritious bran and germ, which provide essential fiber, vitamins, and minerals linked to a reduced risk of chronic disease [1]. Despite these nutritional benefits, the bran and germ components present significant technical challenges in breadmaking. The coarse bran particles physically disrupt the gluten network, while enzymes and lipids from the germ can degrade proteins and destabilize the dough. This interference weakens the dough structure, resulting in poor gas retention and final bread products with a dense, coarse crumb and smaller loaf volume [2].

Therefore, hydrocolloids are incorporated as an additive to overcome these issues and improve the quality of the WWB samples. Hydrocolloids are natural polymers that offer multiple functional benefits, including improving dough performance, enhancing bread characteristics, increasing moisture holding capacity, and upholding sensorial quality throughout storage [3]. Plant-based hydrocolloids are currently a key focus in the bakery industry due to their diverse functional properties. One such unexplored hydrocolloid is Date Palm Gum (DPG), which has emerged as a potential additive. The gum is sourced from *Phoenix dactylifera* (date palm) [4], a tree native to the arid regions. DPG is a complex mixture of polysaccharides, glycoproteins, and other minerals (calcium and potassium). Herein, the polysaccharides, composed of monomers like arabinose, galactose, and rhamnose, provide unique properties of the gum [5,6]. Some of these unique properties encompass a marked water binding ability and pseudoplastic flow behavior [7,8]. This is consistent with the broader nutritional value of date fruit, which is rich in bioactive components with antioxidant and antimicrobial properties. They are also a valuable source of dietary fiber, which can be extracted and used as a value-added ingredient [9].

Despite the perceived potential of natural exudates from date palm, DPG remains a significantly unexplored hydrocolloid. Its specific functional impact in bakery products is largely unknown, and as a novel ingredient, its formal regulatory status and food-grade specifications have not yet been established. This lack of foundational data presents a clear need for a fundamental assessment. Therefore, the current study provides a fundamental investigation by performing an in-depth analysis of the role of DPG at concentrations ranging from 0 to 3% (*w*/*w*) on the textural and physicochemical characteristics of WWB. A range of analytical tools, including microscopy, moisture analysis, colorimetry, impedance spectroscopy, deswelling analysis, FTIR spectroscopy, and texture profile analysis, were used for this purpose.

## 2. Materials and Methods

### 2.1. Materials

Whole wheat flour (WWF) (Brand: Classic Wheat Flour Atta, Make: Avent Agro Private Limited, New Delhi, India) and instant dry yeast (Brand: Annprash Dry yeast powder, Jyoti Trade Corporation, Jaipur, Rajasthan, India) were purchased from Flipkart Grocery. According to information on the packaging label, the nutritional composition of WWF per 100 g is 0.50 g sugar, 11.73 g protein, 4.66 g sodium, and 360 kcal energy. Commercial salt (Brand: TATA Salt, Make: Tata Chemicals Limited, Devbhumi Dwarka, Gujarat, India), sugar (Brand: Mishti by Dhampur, Make: Dhampur Sugar Mills Limited, New Delhi, India), and Rice Bran Oil (RBO) (Brand: Fortune, Make: Adani Wilmar Limited, Ahmedabad, Gujarat, India) were procured from the market. RBO used in this study contains 100 g of fat, 22 g of polyunsaturated fatty acids, 2 g of trans fatty acids, and 50 mg of vitamin E per 100 g. Lastly, Date Palm Gum (DPG) (Brand: Natural Herbs, Make: Sai Herbs, Amritsar, Punjab, India; ~75% purity) was bought from Amazon.

### 2.2. Formulation of Whole Wheat Bread (WWB)

WWB was formulated by using varying amounts of Date Palm Gum (DPG), ranging from 0% to 3% (*w*/*w*). The samples were labeled as D0 (control, 0% *w*/*w* of DPG), D1 (0.5% *w*/*w* of DPG), D2 (1% *w*/*w* of DPG), D3 (2% *w*/*w* of DPG), and D4 (3% *w*/*w* of DPG). Initially, the ingredients specified in Table 1 were added to the bread maker (Model: 16010; Make: KENT RO Systems Ltd., Noida, Uttar Pradesh, India) and set to the “Whole Wheat Bread” program. The mixture initially underwent a 5 min pre-warming phase, followed by alternating kneading (37 min at ~100 rpm) and resting (28 min) cycles. Thereafter, the mixture was subjected to fermentation (35 ± 2 °C) for 120 min. After fermentation, the dough was evenly spread into a cake tin of dimensions 10.18 cm × 10.18 cm × 5.8 cm (L × B × H). It was then baked in an air fryer (Model: IBLAF 650MN, Make: Innovative Technologies, IBELL, Aluva, Kerala, India) at 180 °C for 8 min. After baking, WWB was allowed to cool at room temperature (RT; 25 °C ± 2 °C) for 60 min before further analysis.

In this study, DPG was added by replacing an equivalent weight of water to maintain a constant total hydration level (water + gum) of 100% (*w*/*w*, based on flour weight) for formulations D1–D4. This replacement method was chosen to assess the intrinsic functional impact of the hydrocolloid on the bread system itself.

### 2.3. Surface Topology

A handheld digital microscope (Model: 44308 Celestron Pro Microscope, Make: Celestron LLC, Torrance, CA, USA) was used to observe the surface microstructure of the samples (crust and crumb). Briefly, the samples were cut into small square pieces measuring 2 cm × 2 cm and analyzed to examine their surface attributes [10,11].

### 2.4. Moisture Analysis of Crumb

A digital moisture analyzer (Model: PGB1MB analyzer; Make: Wensar, Chennai, Tamil Nadu, India) fitted with a halogen heating source was used to determine the moisture content of the samples. Approximately 2 g of the bread crumbs were taken and placed in an aluminum pan, which was then heated at 180 °C until a constant weight was reached. The moisture content of the samples was evaluated using Equation (1) [10].

(1) $$MO\left(\%\right)=\frac{W^{\prime }-W\prime \prime }{W\prime }\times 100$$

where *W′* refers to the initial weight of the samples, *W″* refers to the final weight of the samples after heating at 180 °C, and MO (%) denotes the moisture content of the samples in percentage.

### 2.5. Water Activity Analysis

The water activity (aw) of the bread crumbs was measured, as per the method described in [12] with slight modifications.

### 2.6. Colorimetric Analysis

Colorimetric analysis of the samples was performed using a colorimeter developed in the lab to obtain CIELab color parameters. Initially, the colorimeter was calibrated using white and black tiles. The bread sample (square-shaped, dimension: 2 cm × 2 cm) was placed in a 35 mm Petri dish, and then the analysis was performed to determine the lightness (L*), red-green balance (a*), and yellow-blue balance (b*) of the prepared samples in the CIELab color space. Then, the computed color indices whiteness index (*WI*; Equation (2)), yellowness index (*YI*; Equation (3)), and brownness index (*BI*; Equation (4)) were computed [12,13]. Additionally, the CIE1931 chromaticity coordinates (x, y) were also calculated in order to generate the chromaticity diagram. A chromaticity diagram provides a visual map of hue and saturation independent of brightness, enabling objective comparison of sample color quality and differences in pigment formation. Furthermore, the reflectance spectral profiles of the samples were analyzed using an in-house developed reflectance spectrophotometer (Patent Pending, Indian Patent Application No. 202331051781). This analysis provides additional information on how light reflects at specific wavelengths, corresponding to particular chemical compounds (chromophores) formed during baking. Finally, the hyperspectral images of the samples were captured in the wavelength range from 340 nm to 850 nm (Patent Pending, Indian Patent Application No. 202331051781) to qualitatively visualize the spatial distribution of color on the crumb surface [13].

(2) $$WI=100-\sqrt{{\left(100-L^{*}\right)}^{2}+{\left(a^{*}\right)}^{2}+{\left(b^{*}\right)}^{2}}$$

(3) $$YI=142.86\times \frac{b^{*}}{L^{*}}$$

(4) $$BI=\frac{\left\{100\left(X-0.31\right)\right\}}{0.17}$$

where L* is lightness, a* is red-green, and b* is yellow-blue color parameters;

$$X=\frac{\left(a^{*}+{1.75L}^{*}\right)}{\left(5.645\;X\;L^{*}+a^{*}-3.01{2b}^{*}\right)}$$

### 2.7. Electrical Impedance Spectroscopy

An impedance analyzer (Model: Impedance breakout board for Analog Discovery 2, Digilent, National Instrument, Austin, TX, USA) was employed to obtain the impedance profiles of the samples. The WWB samples were cut into cuboidal pieces of dimensions 2.5 cm × 2.5 cm × 3 cm (L × B × H). Subsequently, a set of stainless-steel probes (placed parallel at a distance of 1 cm) was inserted inside the crumb. The impedance measurement was performed over a frequency range of 1 Hz to 1 kHz [13].

### 2.8. Deswelling Analysis

Deswelling profiles of the WWB samples were evaluated by taking uniform crumb cubes cake tin of dimensions 10.18 cm × 10.18 cm × 5.8 cm (L × B × H), placed in Petri dishes and dehydrated at 60 °C using a food dehydrator (Model: AGARO Regal; Serial No: FD24050790; Manufacturer: Universal Corporation Limited, Kolkata, India). The first set of measurements was taken before dehydrating the samples, followed by sequential weight measurements at 15 min time intervals for 2 h and at 30 min intervals over the subsequent 3 h. The deswelling index (DS%) was calculated using Equation (5)

(5) $$\mathrm{DS}\;(\%)=\frac{W_{i}-W_{t}}{w_{i}}\times 100$$

where W_i_ is the initial weight (before deswelling study) and W_t_ is the final weight (after the deswelling study) of the WWB samples.

### 2.9. FTIR Spectroscopy

The IR absorption spectra of the WWB samples were measured using an ATR-FTIR spectrophotometer (Alpha-E; Bruker, Billerica, MA, USA), which was equipped with an attenuated total reflectance (ATR) module with a zinc selenide (ZnSe) crystal. The bread samples were measured in the range of 4000–400 cm^−1^, and each sample was analyzed using 25 scans and recorded at a spectral resolution of 4 cm^−1^ [14].

### 2.10. Textural Analysis

Texture properties of WWB samples (hardness, cohesiveness, springiness, and resilience) were analyzed using a texture analyzer (Model: Texture Analyzer HD Plus; Stable Micro Systems, Godalming, UK) equipped with a 35 mm flat probe. The uniform cubes 1.5 cm × 1.5 cm × 1.5 cm (L × B × H) were subjected to two consecutive compression cycles at a level of 50% strain and 1 mm/s speed within 5 s between cycles. Further, to analyze stress relaxation, the samples of the same dimensions as cubes were compressed by 5 mm at a speed of 1 mm/s after a force of 5 g was achieved. The probe was held in place for 60 s to observe the force reduction before the probe was released and allowed to return to its original position [12,15].

### 2.11. Total Phenolic Content Determination

The total phenolic content (TPEC) of bread samples was determined using the Folin–Ciocalteu method as elucidated in [16] with minor modifications. Subsequently, the results have been represented as mg gallic acid equivalent (GAE)/g.

### 2.12. Statistical Analysis

Experiments were performed in triplicate, and results are reported as mean ± standard deviation. Statistical analysis was performed using JMP PRO software (Version 18, JMP Statistical Discovery LLC, Cary, NC, USA). One-way analysis of variance (ANOVA) was used to detect the significant differences among the parameters, followed by post hoc Tukey tests. Spearman correlation analysis was conducted with OriginPro Software (Version: 2020b, Company: OriginLab Corporation, Northampton, Massachusetts, United States) to assess the statistical significance among the profiles.

## 3. Results and Discussions

### 3.1. Visual and Physical Analysis

**Figure 1:**
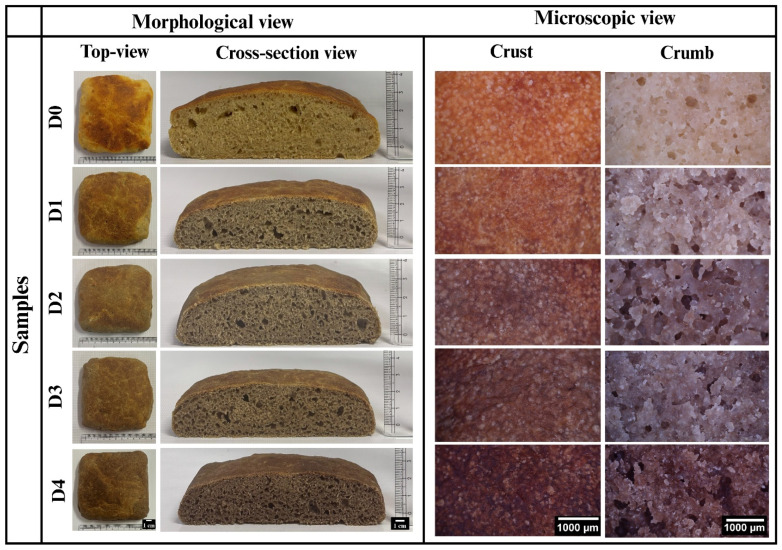
Morphological and microscopic view of WWB samples containing date tree gum (DTG) in different concentrations: D1 (0.5% *w*/*w* of DTG), D2 (1% *w*/*w* of DTG), D3 (2% *w*/*w* of DTG), and D4 (3% *w*/*w* of DTG).

After the baking process was completed, the WWB samples were cooled to room temperature for 1 h. This was done to ensure that the samples would be at an appropriate temperature for handling. The overall visual appearance and cross-sectional morphology of all bread samples are shown in Figure 1. As shown in Figure 1, D0 (control sample; without DPG) exhibited a distinct combination of yellowish and brownish regions. The yellow color in some regions was due to carotenoid pigments such as β-carotene, lutein, and zeaxanthin, which are naturally present in wheat flour [17,18]. The brown coloration was due to two nonenzymatic browning reactions: the Maillard reaction (MR) and caramelization. The MR occurs between amino groups from proteins and reducing sugars, while caramelization is the pyrolytic breakdown of sugars at high temperatures. Both processes produce melanoidins and other compounds responsible for the deep brown color and roasted flavor [19,20]. The incorporation of DPG (0.5% *w*/*w*) in D1 induced the formation of a light brown crust with a rough and uneven surface. This color change might be attributed to the inherent brown pigmentation of the gum, primarily due to the presence of polyphenolic compounds such as tannins. With the increasing concentration of DPG in D2, a uniform, medium-brown crust with a smoother surface was observed, compared to D1. This might be attributed to DPG retaining more moisture, thereby facilitating the even distribution of heat and resulting in consistent browning. Moreover, D3 and D4 displayed darker brown crusts with a rougher surface compared to D2. The increased roughness likely results from the excessive binding of DPG with water due to the higher concentration of gum, which limits its availability for proper starch gelatinization and gluten hydration, thus forming a textured surface [21].

The cross-sectional morphology of the bread samples in Figure 1 demonstrated the matrix integrity, porosity, and density of the bread crumbs. D0 (control, without DPG) showed a dense crumb with few large, irregularly distributed air pockets, indicating poor aeration and compact internal structure [22]. With the incorporation of DPG, D1 exhibited a slightly open crumb with small and large pores unevenly distributed, indicating improved aeration compared to D0 [23]. D2 showed well-aerated crumb with medium-sized air pockets that are evenly distributed, suggesting improved fermentation that contributes to better leavening. This could be due to the formation of a strengthened network of gluten-starch-gum at this concentration, resulting in a more structured and robust crumb structure [24]. Further, D3 showed pores of varying sizes distributed non-uniformly, which might be due to DPG interfering with water mobility and gas diffusion, resulting in uneven gas entrapment. However, D4 showed a dense crumb with an increased number of small, evenly distributed pores. This was likely due to subdued fermentation activity [25], as the higher level of DPG might have limited water availability, reducing gas generation and crumb development [21].

### 3.2. Microscopic Evaluation

Microscopic analysis helps to evaluate the microstructural features of the food products, providing visual insights into the surface morphology, pore distribution, and matrix organization at the microscopic level [26]. Figure 1 illustrates the crust surface characteristics of the WWB samples. As depicted in Figure 1, D0 showed a comparatively smooth and mildly brown crust with white, sparse, small, shiny granules. The browning indicated MR and caramelization [27], while the granules formed were likely due to starch gelatinization and retrogradation, a process where the starch chains reassociate into a semi-crystalline structure during cooling [28]. Incorporation of DPG in D1 and D2 led to an increased number of granules with a comparatively rougher and darker crust surface, indicating improved MR and caramelization [27]. Interestingly, while the microscopic inspection revealed a rougher surface of D2, it appeared smoother during visual analysis. This might be attributed to the high water-holding capacity of DPG, which retains moisture on the surface and results in a visibly smoother crust. However, the polymer chains of DPG might physically interfere with the uniform swelling of starch granules at the surface. Consequently, a microscopically rougher yet well-integrated granular surface structure was produced [29]. In D3, the crust appeared dark brown with an even rougher texture and evenly distributed granular structure. In D4, the crust exhibited the highest level of browning with further increase in surface roughness and a dense distribution of smaller granular structure. The roughness observed in D3 and D4 might be a result of DPG’s high water-binding capacity, which limits starch gelatinization and gluten hydration, as corroborated by visual inspection. In addition, the increased browning is due to enhanced MR [27] and the intrinsic color of DPG due to its high concentrations [30].

The micrographs of the crumb surface showed significant variation in pore size distribution, matrix cohesiveness, gas retention, and baking-related thermal behavior, as presented in Figure 1. D0 exhibited a compact and dense crumb matrix with small to medium-sized pores. Herein, the yellowish-brown appearance indicated minimal Maillard browning, characteristic of typical WWB crumb color [31]. Initially, D1 exhibited a comparatively darker crumb color and a considerable increase in pore size compared to D0. Such observations indicated better gas retention due to effective yeast fermentation [32], which released carbon dioxide within the gluten-starch matrix, causing dough expansion and leading to improved crumb aeration. D2 showed a further increase in pore size. This could be due to its highly structured and strengthened crumb matrix, which facilitated the dough expansion and gas retention, forming a more porous crumb [22]. In contrast, D3 exhibited a restructuring of the crumb network, characterized by a compact crumb structure and a smaller pore size than D2. Additionally, the crumb color was darker than D1 and D2, due to increased Maillard activity and an increased amount of DPG. Finally, D4 showed the most intense brown coloration, irregular air cells, and less distributed matrix. The compact crumb structure observed in D3 and D4 might be attributed to the limited water availability for proper gluten hydration and yeast activity due to the high water-binding property of DPG, which interferes with the fermentation process and proper gas diffusion. Thus, a less porous crumb with reduced gas retention is formed. Additionally, the combined effects of advanced Maillard browning [27] and the inherent brown coloration of DPG might be responsible for the excessive browning.

### 3.3. Moisture Content Analysis

**Figure 2:**
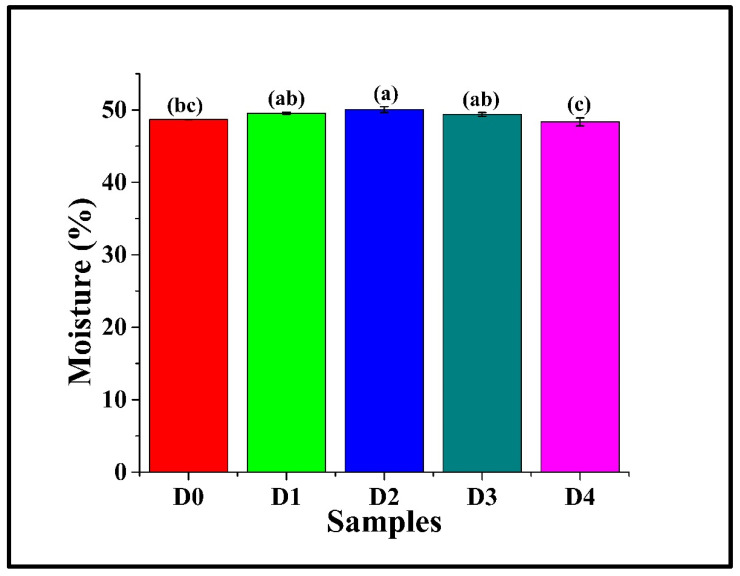
Graph representing the moisture content of WWB samples containing varying concentrations of DTG: D0 (control, absence of DTG), D1 (0.5% *w*/*w* DTG), D2 (1.0% *w*/*w* DTG), D3 (2.0% *w*/*w* DTG), and D4 (3.0% *w*/*w* DTG). The data are presented as mean ± standard deviation. Different letters denote statistical differences among the samples (*p* < 0.05).

Moisture analysis in bread samples is an important parameter, as moisture content directly impacts key quality attributes like texture (softness, crumb structure), shelf-life (freshness, staling, microbial spoilage), and ultimately, consumer acceptance. It was observed that the moisture content of D0 was 48.68 ± 0.01%, as in Figure 2. Incorporation of DPG in D1 (49.54 ± 0.14%), D3 (49.39 ± 0.25%), and D4 (48.35 ± 0.55%) (*p* > 0.05) showed statistically similar moisture content compared to D0, but significantly lower than D2 (50.06 ± 0.41%) (*p* < 0.05). This may be attributed to DPG, which, at 1% effectively enhanced water retention within the gluten-starch matrix, forming hydrogen bonding between the hydroxyl group and water molecules, reducing moisture loss during baking. Similarly, Borchani et al. (2011) reported that incorporating date flesh fiber concentrate (DFFC) into wheat bread enhanced dough water absorption, which they attributed to the hydroxyl-rich structure of the fiber [31,33]. Among the DPG-containing samples, D1, D2, and D3 had similar moisture content, though D2 exhibited a subtly higher value. However, D4 displayed the lowest moisture content (*p* < 0.05). The observed reduction in D4 is likely due to the strong binding of the available water molecules with the DPG molecules, which limited the water availability for starch gelatinization and gluten hydration [34].

### 3.4. Analysis of Water Activity

**Figure 3:**
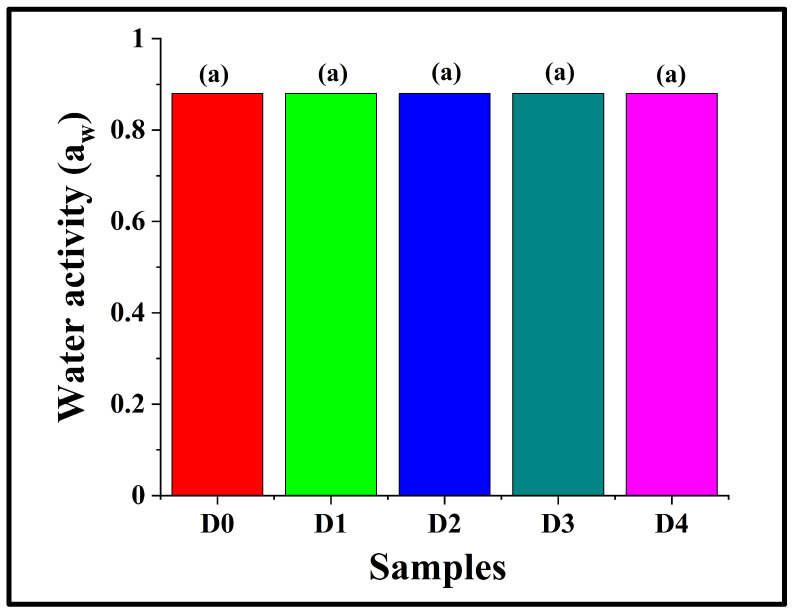
Graph representing the water activity of WWB samples containing varying concentrations of DPG: D0 (control, absence of DPG), D1 (0.5% *w*/*w* DPG), D2 (1.0% *w*/*w* DPG), D3 (2.0% *w*/*w* DPG), and D4 (3.0% *w*/*w* DPG). The data are presented as mean ± standard deviation. Different letters denote statistical differences among the samples (*p* < 0.05).

Water activity (aw) is a critical parameter for food quality and stability, as it influences the texture and shelf life of the product [35]. Herein, it was observed that the aw values of all the bread samples were 0.88 ± 0.00, despite an increment in the moisture content of D2 as compared to D0 (Section 3.3), thereby indicating that the addition of DPG did not affect the water activity of the crumb matrix (*p* > 0.05). This might be attributed to the fact that the additional water molecules in D2 and other DPG-containing breads remained bound within the hydrocolloid-starch–protein network rather than contributing to the free-water portion (Figure 3). These observations are consistent with the findings reported by Liu et al. (2017), wherein the authors observed that the addition of hydrocolloids (konjac glucomannan, arabic gum, hydroxylpropylmethylcellulose, and apple pectin) in breads made of potato-wheat flour showed no marked changes in the water activity values, but significant alterations in the water content [34,36]. Collectively, the results indicate that DPG altered the moisture retention properties of the bread samples, without compromising their microbial safety or physicochemical stability.

### 3.5. Colorimetric Analysis

#### 3.5.1. Analysis of the Color Parameters

**Figure 4:**
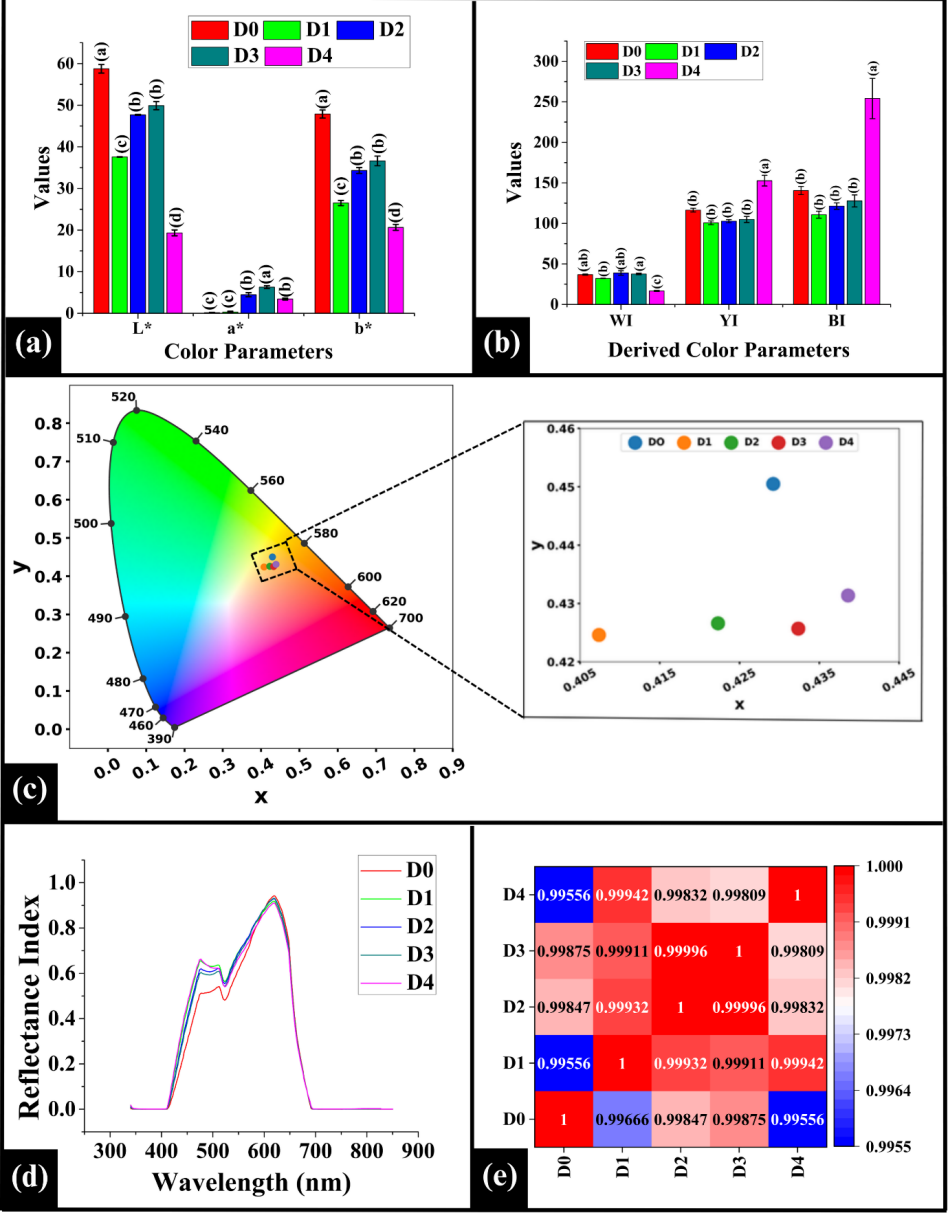
Graph representing color parameters of WWB samples (**a**) L*, a*, and b*; (**b**) calculated color indices: parameters: whiteness index (WI), yellowness index (YI), and brownness index (BI), (**c**) CIE chromaticity diagram (**d**) line graph representing the reflectance profiles of WWB samples, and (**e**) heatmap depicting Spearman correlation pf reflectance profiles. The data are presented as mean ± standard deviation. Different letters denote statistical differences among the samples (*p* < 0.05).

In this study, the CIELAB color parameters (L*, luminosity; a*, red-green; b*, yellow-blue) were analyzed to assess the crumb color that helps elucidate the influence of varying DPG concentrations on Maillard browning and caramelization in WWB [37]. Herein, D0 (control sample) displayed an L* value of 58.78 ± 1.04, as depicted in Figure 4a. Incorporation of DPG resulted in significantly lower L* values in all the samples, i.e., D1 (37.24 ± 0.40), D2 (46.69 ± 0.13), D3 (47.42 ± 1.59), and D4 (17.23 ± 0.71) (*p* < 0.05), suggesting a darkening in the crumb coloration. Among DPG-containing samples, D1 showed significantly lower L* value than D2 and D3 but higher than D4 (*p* < 0.05). This darkening in D1 can be associated with the combined effects of the oxidation of polyphenolic compounds [38] that are present in DPG (into brown compounds like quinones) [39] and enhanced Maillard browning [40]. D2 exhibited significantly higher L* value than D4 (*p* < 0.05) but was statistically similar to D3 (*p* > 0.05). The increase in the L* value in D2 and D3 could be attributed to a more uniform distribution of color-forming compounds at these DPG levels (1% *w*/*w* and 2% *w*/*w*), within the crumb matrix. This might have led to a better overall light reflection and thus an overall lighter appearance. However, D4 exhibited the lowest L* value among all the samples (*p* < 0.05), suggesting maximum darkening. This might be due to the reaction of abundant polysaccharides and phenolic compounds from DPG with dough components, which resulted in pronounced MR and caramelization reaction. Additionally, the lower moisture content and the denser crumb possibly restricted the light scattering, leading to further darkening [41].

Regarding a*, D0 displayed a value of 0.25 ± 0.11 as in Figure 4a. Upon incorporation of DPG in D1 (0.72 ± 0.18), a similar a* value compared to D0 (*p* > 0.05) was observed. This indicated that the redness in D1 remained similar to that of the control due to a lower concentration of DPG (0.5%) and limited MR. Further addition of DPG in D2 (3.68 ± 0.55), D3 (6.11 ± 0.63), and D4 (3.25 ± 0.48) showed a significantly higher a* value than D0 (*p* < 0.05). The increase in a* values in these samples likely reflects enhanced oxidation of DPG-derived polyphenols and an intensified MR, resulting in the formation of increased amounts of reddish-brown pigments [42]. Similar observation was reported by Liu et al. (2017), where the addition of apple pectin (AP) (2% *w*/*w*) and hydroxypropyl methylcellulose (HPMC) (2% *w*/*w*) in potato-wheat bread led to a significant increase in the a* value [34,36]. Among the DPG-fortified samples, D1 exhibited a significantly lower a* value than D2, D3, and D4 (*p* < 0.05). D2 exhibited a lower a* value than D3 but was statistically similar to D4 (*p* > 0.05). Moreover, D3 recorded the highest a* value within the DPG-containing samples (*p* < 0.05).

The positive b* value, as illustrated in Figure 4a, can be primarily attributed to the natural presence of yellow carotenoid pigments in wheat flour, such as lutein, β-carotene, and zeaxanthin [43]. Herein, a similar trend was observed to that of L*, where DPG incorporation resulted in lower b* values, reflecting a darker color of the crumb. This reduction can be explained by the enhanced MR and phenolic oxidation, which led to the formation of darker pigments such as melanoidins [44] and quinones [45]. These pigments likely masked the natural yellow coloration of the wheat carotenoids, thereby lowering the b* value. Resultantly, an overall darker crumb was observed that is consistent with the lower L* values.

The values of L*, a*, and b* were used to calculate the derived color indices, i.e., the whiteness index (WI), yellowness index (YI), and brownness index (BI) of the WWB samples [11]. In the case of WI, D0 displayed a value of 36.50 ± 0.36, as in Figure 4b. Incorporation of DPG in lower amounts in D1 (31.53 ± 0.72), D2 (34.88 ± 2.22), and D3 (38.29 ± 3.11) did not significantly alter the WI values compared to D0 (*p* > 0.05), indicating that the perceived brightness of these samples is similar [13]. This could be attributed to the non-significant change in the L*, a*, and b* values among the samples, which did not alter the overall WI values [13,14]. However, D4 (16.67 ± 1.55) exhibited a significantly lower WI value than D0 (*p* < 0.05), which is consistent with its significantly lower L* and b* values. Among the DPG-fortified samples, the WI value of D1 was statistically similar to D2 (*p* > 0.05), but considerably lower than that of D3 (*p* < 0.05), while higher than D4. The WI value of D2 and D3 was statistically similar (*p* > 0.05), but higher than D4, which exhibited the lowest WI value (*p* < 0.05).

In the case of YI, D0 exhibited a value of 117.23 ± 1.00, as illustrated in Figure 4b. After the addition of DPG, the YI values of D1 (101.96 ± 3.98), D2 (109.82 ± 8.34), and D3 (103.60 ± 8.84) were statistically similar to D0 (*p* > 0.05), but lower than D4 (*p* < 0.05). The similarity in D1, D2, and D3 could be attributed to the proportional reduction in their L* and b* values, which maintained an equivalent YI value of the samples. This implies that the overall perceived yellowness among them remains similar, despite the darkening of the bread crumb [46]. Among the DPG-added samples, the YI values of D1, D2, and D3 were statistically similar (*p* > 0.05). However, D4 exhibited the highest YI value among all the samples (*p* < 0.05). It indicated an increased yellowness perception in D4, despite having the lower b* values. Herein, the substantial decrease in the L* value offset the decrease in b* value, resulting in a higher overall YI value.

A similar trend was observed in BI values as that of YI values, where D0–D3 were statistically similar and D4 exhibited the highest BI value, as shown in Figure 4b. This was likely due to progressive polymerization of melanoidins and oxidation of phenolic compounds into o-quinones [45], forming yellow-brown polymers, which proportionally increased the BI values in D4. This aligned with its lower L* and b* values.

#### 3.5.2. Analysis of Chromaticity

Chromaticity provides information on the hue and saturation of color; however, it fails to provide information on the brightness of the samples. The chromaticity diagram is a horseshoe-shaped spectrum that represents spectral colors in the wavelength range of ~380 nm (violet) and 700 nm (red) [47]. It can be observed that the color coordinates (x, y) of the WWB samples were closely clustered: D0 (0.425, 0.455), D1 (0.410, 0.425), D2 (0.420, 0.430), D3 (0.430, 0.430), and D4 (0.440, 0.435), as shown in Figure 4c. These color coordinates were located within the yellow-orange region of the chromaticity diagram, which is generally associated with bakery products. Although the difference in hue and saturation was minor, a gradual shift of the coordinates was observed from D1 to D4. This shift, together with the reduction in L* values observed earlier, indicates that an increase in the DPG concentration might enhance the MR and caramelization [48] in the bread samples.

#### 3.5.3. Analysis of the Reflectance Index

The Reflectance Index (RI) is a quantitative measure of the light reflected from the surface at specific wavelengths within the visible spectrum, providing crucial insights into visual attributes like crumb luminosity, surface appearance, and color uniformity in the WWB samples. Reflectance spectra of bread crumb samples (D0–D4) exhibited a distinct pattern across the visible region (300–900 nm), indicating the influence of DPG on the crumb brightness, as depicted in Figure 4d. Notably, the spectra revealed an initial peak at 477 nm, followed by a broader peak at 513 nm, a dip at 523 nm, and an intense peak at 619 nm. The sharp peak at 477 nm, located within the blue-violet region, might be due to the absorption of compounds like carotenoids (e.g., lutein and zeaxanthin), which are naturally present in the wheat germ and bran [49]. These compounds naturally absorb in the blue-violet region, conferring a yellowish hue in the crumbs. Following this, the broader peak at 513 nm is associated with the green region of the visible spectrum. This suggests the presence of intermediate browning compounds from the MR, including reductones and Amadori compounds, which absorb light in the 500–520 nm range [50]. The dip observed at 523 nm, associated with the yellow-green region, was likely due to absorption by wheat phenolic compounds, such as ferulic acid, caffeic acid, and flavonoids, whose conjugated structures may have reduced reflectance. The intense peak at 619 nm is associated with the orange-red region and might be due to the accumulation of high-molecular-weight melanoidins [51]. These compounds, formed during the final stage of MR due to prolonged heating, strongly reflect light in this region, contributing to the orange-red coloration [52].

The Spearman correlation was employed to compare the shape of the reflectance profiles, which is independent of their overall magnitude, as depicted in Figure 4e. Herein, the high Spearman correlation values (ρ = 0.99556 to 0.99996) imply that the spectral shapes were nearly identical. This clearly indicates that the incorporation of DPG did not significantly alter the fundamental spectral pattern. The consistent spectral pattern among the samples indicates that the MR and the caramelization process [53], driven by DPG, impacted the overall intensity, instead of markedly changing the hue.

**Figure 5:**
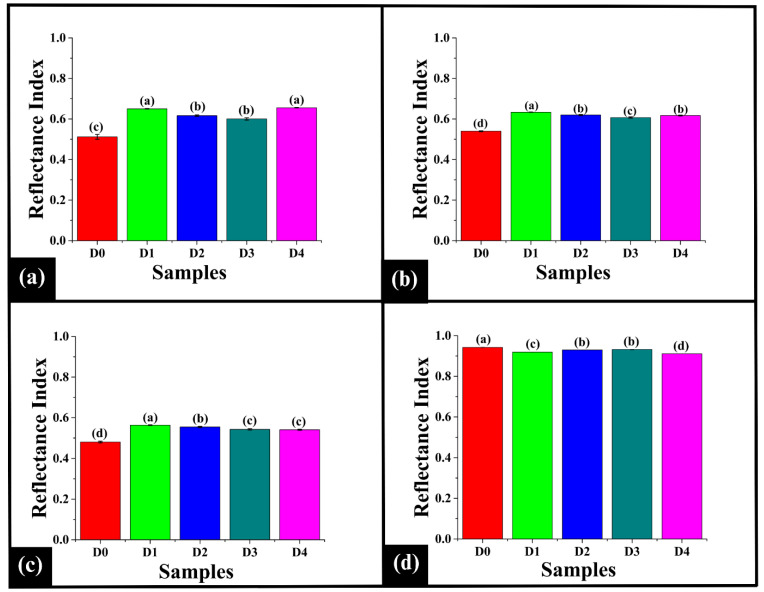
Bar graph representing distinct reflectance peaks for prepared WWB samples (D0–D4). (**a**) 477 nm, (**b**) 513 nm, (**c**) 523 nm, and (**d**) 619 nm. The data are presented as mean ± standard deviation. Different letters denote statistical differences among the samples (*p* < 0.05).

Further, the reflectance index was analyzed at the identified wavelengths, including 477 nm, 513 nm, 523 nm, and 619 nm. As shown in Figure 5a, at 477 nm, D0 exhibited the RI477 value of 0.51 ± 0.01. Incorporation of DPG results in an increase in the RI_477_ value of D1 (0.65 ± 0.00), D2 (0.62 ± 0.00), D3 (0.60 ± 0.01), and D4 (0.66 ± 0.00) (*p* < 0.05). Among the DPG-added samples, D1 showed a significantly higher RI_477_ value than D2 and D3 (*p* < 0.05). Furthermore, the RI_477_ value of D1 was statistically similar to D4 (*p* > 0.05). In contrast, the RI_477_ values of D2 and D3 were observed to be statistically comparable to each other (*p* > 0.05) and were significantly lower than that of D4 (*p* < 0.05). The results exhibited an increase in the RI_477_ values of all the samples after the incorporation of DPG, indicating a reduction in the absorption of blue-violet light. This could be attributed to the physical entrapment of the carotenoid pigment [54] by the inherent polyphenolic compounds present in DPG [55], which lowered the presence of free pigments available for light absorption. These results align with the observed b* values of the samples, which were lower than those of the control.

At 513 nm, D0 exhibited the lowest RI_513_ value of 0.54 ± 0.00 (*p* < 0.05), as depicted in Figure 5b. Upon addition of DPG in D1, the highest RI513 value of 0.634 ± 0.00 was observed (*p* < 0.05). An increase in the DPG content resulted in a decrease in the RI_513_ value in D2 (0.620 ± 0.00) and D3 (0.607 ± 0.00) (*p* < 0.05). A further increase in the DPG content in D4 resulted in an increase in the RI513 value (0.617 ± 0.00), which was similar to D2 (*p* > 0.05).

Thereafter, at 523 nm, D0 showed the lowest RI_523_ value of 0.48 ± 0.00 (*p* < 0.05), as presented in Figure 5c. Upon addition of DPG in D1, the RI_523_ value (0.56 ± 0.00) was the highest (*p* < 0.05). As mentioned above, improved water retention and optimal crumb aeration might have enhanced the light scattering [56], thereby increasing the reflectance at the wavelength. A decline in the RI_523_ value was observed from D2 (0.55 ± 0.00) to D4 (0.54 ± 0.00) compared to D1 (*p* < 0.05). The RI523 value D4 was statistically similar to D3 (0.54 ± 0.00) (*p* > 0.05).

Based on the results, the addition of DPG resulted in increased RI_513_ and RI_523_ values in the samples compared to the control (D0). This generally indicates reduced pigment absorption in the yellow-green region, resulting in a lighter crumb. However, the DPG-containing samples exhibited an increased browning. The color shifts at 513 and 523 nm signified the dominance of MR products in baked systems [57]. These wavelengths are commonly associated with green and yellow pigments in botanical samples [58]. However, in the case of baked products, they imply the reflection of green and yellow-green light by newly formed, light-absorbing chromophores [59]. This is consistent with the lower L* values and higher a*, indicating a shift towards a darker, more saturated yellow-red crumb coloration.

Lastly, at 619 nm, D0 displayed the RI_619_ value of 0.94 ± 0.00, which was significantly higher than the DPG-added samples (*p* < 0.05), as shown in Figure 5d. Among the DPG-containing samples, the RI619 value of D2 (0.930 ± 0.00) and D3 (0.93 ± 0.00) was higher than D1 (0.919 ± 0.00) and D4 (0.91± 0.00). However, D2 and D3 were statistically similar (*p* > 0.05). The RI_619_ value of D1 was significantly higher than that of D4 (*p* < 0.05). Overall, the results displayed decreased R_I619_ values with the addition of DPG in the samples, indicating an increase in absorption in the orange-red region. This could be attributed to the reaction of additional polysaccharides of DPG with the dough components, which intensifies the MR [60], leading to greater accumulation of melanoidins [61]. This also aligned with the lower values L* and b* in the samples.

#### 3.5.4. Hyperspectral Imaging

Hyperspectral imaging (HSI), which provides both spatial and spectral information, is widely used in the bakery industry for the nondestructive evaluation of food structure, composition, moisture content, and ingredient uniformity [62]. At 477 nm, D0 exhibited a mixture of dark blue, light blue, and green pixels, as shown in Figure 5. The dark blue areas indicated high absorbance and lower reflectance, which is associated with a compact or less porous crumb. In contrast, the light blue and green pixels represented moderate to high reflectance. D1 and D2 showed green and yellow pixels reflecting an increase in the reflectance. D3 exhibited green, light blue, and some yellow pixels scattered, indicating lower absorbance and higher reflectance. Furthermore, D4 represented the most intense color, with green and some yellow pixels, reflecting minimal absorbance.

At 513 nm, D0 appeared predominantly green, interspersed with blue pixels, indicating high reflectance with some areas of lower intensity, as depicted in Figure 5. D1 and D2 showed uniform green coloration, suggesting higher reflectance. At D3, green coloration with some light blue pixels was observed, showing spatial variability in reflectance. D4 had bright areas of green with negligible light blue pixels, reflecting a higher localized reflectance. Concerning 523 nm, the reflectance intensity decreased across all the samples, as evident from Figure 5. D0 displayed a mixture of light green, light blue, and dark blue pixels, suggesting moderate reflectance with some areas of high absorbance. Further, D1 and D2 showed a similar pattern to D0, but increased dark blue pixels, indicating localized higher absorbance. D3 exhibited light blue pixels with clusters of dark blue and green pixels, where light blue and green pixels indicated higher reflectance zones and dark blue pixels suggested localized absorbance, signifying a heterogeneous crumb structure. Furthermore, at D4, the predominance of light blue and green pixels was observed with a lower intensity of dark blue pixels, reflecting high reflectance and minimal absorbance regions.

**Figure 6:**
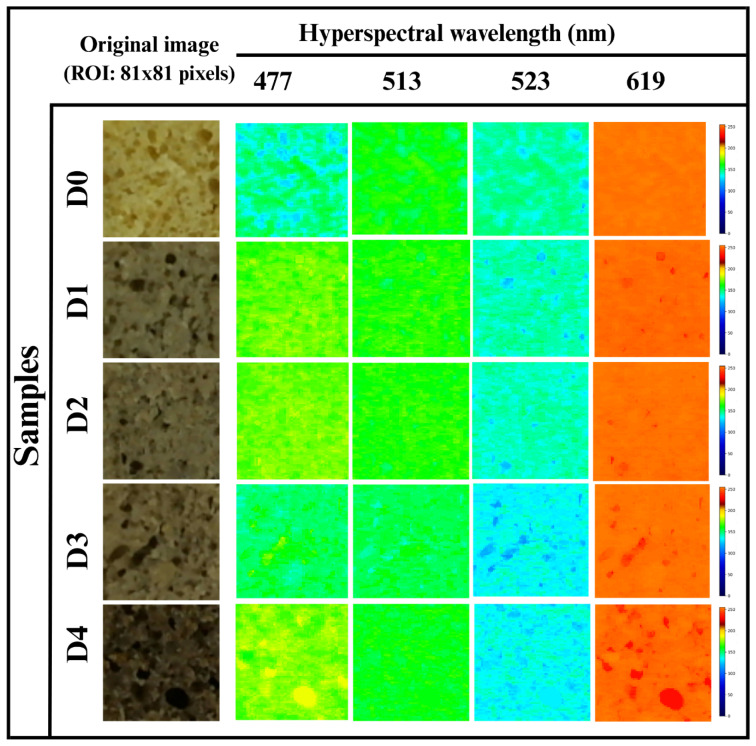
Hyperspectral images of the WWB samples at 477 nm, 513 nm, 523 nm, and 619 nm wavelengths.

At 619 nm, D0 showed prominent orange pixels, suggesting uniformly higher reflectance and lower absorbance, as in Figure 6. D1 and D2 exhibited a mixture of orange and red pixels. The occurrence of a few red pixels indicated a localized increase in reflectance, possibly due to the presence of dense pores. Further, in D3, an orange region with increased red clusters was observed, which indicated high reflectance and reduced absorbance in these regions. Lastly, in D4, pronounced orange regions along with increased red pixels were observed, suggesting relatively higher reflectance in specific areas, indicating a heterogeneous or highly porous crumb structure.

### 3.6. Electrical Impedance Spectroscopy

**Figure 7:**
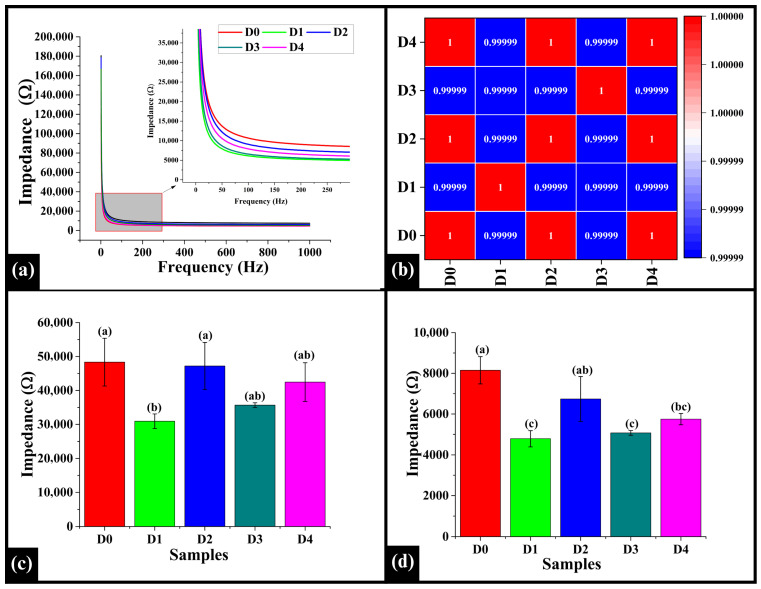
(**a**) Graphs illustrating impedance profile of WWB samples containing DTG, (**b**) heatmap of Spearman correlation coefficients of impedance profiles, (**c**) bar graph representing the low-frequency region, and (**d**) bar graph of the high-frequency region. The data are presented as mean ± standard deviation. Different letters denote statistical differences among the samples (*p* < 0.05).

Electrical Impedance Spectroscopy (EIS) is a nondestructive technique used to assess bread’s moisture content, internal structure, porosity, and overall quality by measuring its electrical response to an alternating current over a range of frequencies [63]. The line graph (Figure 7a) illustrates that all the samples exhibited a decreasing impedance trend as the frequency increased. Notably, at approximately 300 Hz, a decrease was observed in the impedance values, and the curves reached a plateau, indicating that the impedance values stabilized and became constant at higher frequencies. Herein, among the samples, D0 showed the highest impedance value, indicating less conductive behavior, while D1 showed the lowest impedance, suggesting the most conductive pattern. However, D2, D3, and D4 exhibited intermediate impedance values. In Spearman correlation, the values ranged from 0.99999 to 1.00000, indicating a robust correlation between the samples (D0 to D4), suggesting that the addition of DPG did not alter the overall electrical response profile of the samples, as shown in Figure 7b.

Further, the average impedance values in the low-frequency range (1–200 Hz) and end-frequency range (200–1000 Hz) were evaluated for all the samples. The average impedance value in low-frequency, D0 (48,344.55 ± 7040.72 Ω), was observed to be significantly higher than D1 (30,918.17 ± 2154.92 Ω) (*p* < 0.05), but statistically similar to D2 (47,210.97 ± 6930.32 Ω), D3 (35,667.55 ± 686.06 Ω), and D4 (42,461.42 ± 5704.95 Ω) (*p* > 0.05), as presented in Figure 7c. Among the DPG-added samples, the impedance value of D1 was statistically similar to D3 and D4 (*p* > 0.05), but significantly lower than D2 (*p* < 0.05). The impedance value of D2 was slightly higher, but it was still statistically similar to D3 and D4 (*p* > 0.05). Moreover, in the high-frequency range, D0 (8149.74 ± 668.92 Ω) exhibited significantly higher impedance value than D1 4787.36 ± 393.07 Ω), D3 (5071.07 ± 114.16 Ω), and D4 (5748.72 ± 275.99 Ω) (*p* < 0.05), but was statistically similar to D2 (6738.74 ± 1102.30 Ω) (*p* > 0.05), as shown in Figure 7c. Among the DPG-containing samples, D1 remained statistically similar to D3 and D4 (*p* > 0.05), while significantly lower than D2 (*p* < 0.05). The impedance value of D2, even though slightly higher, was statistically similar to D4 (*p* > 0.05), but substantially higher than D3 (*p* < 0.05).

As per the results, the addition of DPG displayed a non-linear effect on the impedance values in both the low and high-frequency ranges. In D1, as corroborated by microscopy results, the aerated crumb structure might have promoted better ionic movement, leading to a decreased impedance value. However, D2 exhibited a higher impedance value, likely due to forming a stronger and more structured matrix, corroborated by microscopic results. This might be attributed to improved starch-gum interactions, which immobilized water and restricted ion mobility, thus increasing electrical resistance [64]. Due to excess water-binding capacity at higher DPG concentrations (D3 and D4), the gum might have led to the disruption of structural integrity and formed a denser crumb matrix, consistent with the microscopic evaluation. Such a disrupted structure might facilitate the release of water from the gel matrix during baking, resulting in increased availability of mobile water molecules and ions in the crumb microstructure [3]. Thus, the impedance values decreased due to increased electrical conductivity in the matrix.

### 3.7. Deswelling Analysis

**Figure 8:**
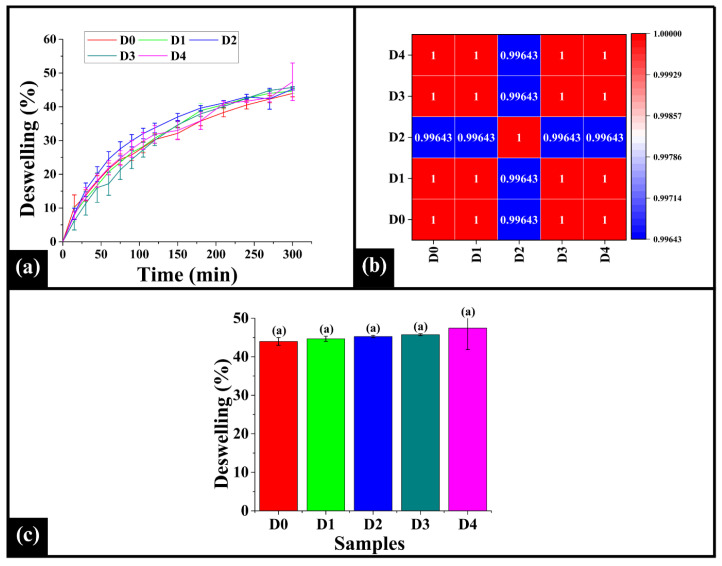
(**a**) Graph representing deswelling profiles of WWB samples, (**b**) heatmap of Spearman correlation coefficient of deswelling profiles, and (**c**) bar graph of deswelling % of the samples. The data are presented as mean ± standard deviation. Different letters denote statistical differences among the samples (*p* < 0.05).

Deswelling analysis assesses crumb texture, staling, and shelf-life by providing information on moisture loss and structural shrinkage, with a lower percentage indicating improved matrix adhesion and uniform crumb structure and a higher percentage associated with increased moisture mobility and an open crumb structure [65]. In contrast, a higher deswelling percentage is associated with increased moisture mobility and open crumb structure. Upon analysis, the deswelling curve (Figure 8a) showed a progressive increase in the deswelling percentage over time. A rapid moisture loss occurred in the initial phase (0–150 min), indicating the release of loosely bound water; thereafter, in the stabilization phase (150–300 min), the deswelling curves reached a plateau phase, suggesting a slower release of tightly bound moisture as equilibrium was approached. Further, the control sample D0 showed the lowest deswelling percentage, indicating better moisture retention, improved matrix cohesion, and a denser crumb structure. Among the DTG-added samples, D1, D3, and D4 displayed an intermediate deswelling percentage, suggesting that higher DTG concentration may not substantially alter water mobility. On the contrary, D2 exhibited relatively higher deswelling percentage, indicating increased moisture mobility and a more open crumb structure that might contribute to enhanced softness and improved sensory characteristics. This finding aligns with the moisture content analysis mentioned in Section 3.3. The Spearman correlation analysis of the deswelling profiles (Figure 8b) showed strong similarity in deswelling among all the samples. The correlation values range from 0.99643 to 1.00000, with D2 exhibiting slightly lower coefficient values in its correlation with D0, D1, D3, and D4, indicating a distinct moisture loss pattern compared to other samples. It could be attributed to stable and structured matrix formation, as observed in microscopic inspection, which supports effective water retention. This stable crumb microstructure might have caused more resistance to water release compared to other samples, resulting in a slower initial deswelling rate. The impedance results also corroborate this particular observation.

Furthermore, the bar graph representing the (%) deswelling (Figure 8c) was analyzed to assess the water retention properties of the samples. The (%) deswelling of D0 (43.97 ± 1.01%), D1 (44.66 ± 0.69%), D2 (45.26 ± 0.29%), D3 (45.74 ± 0.25%), and D4 (47.43 ± 5.56%) was observed to be statistically similar (*p* > 0.05). This observation indicates the total moisture released from the crumb structure was similar, suggesting that the capacity for water retention in the overall bread matrix is not significantly altered by the addition of DPG under experimental conditions [66].

### 3.8. FTIR Spectroscopy

#### 3.8.1. FTIR Profile Analysis

**Figure 9:**
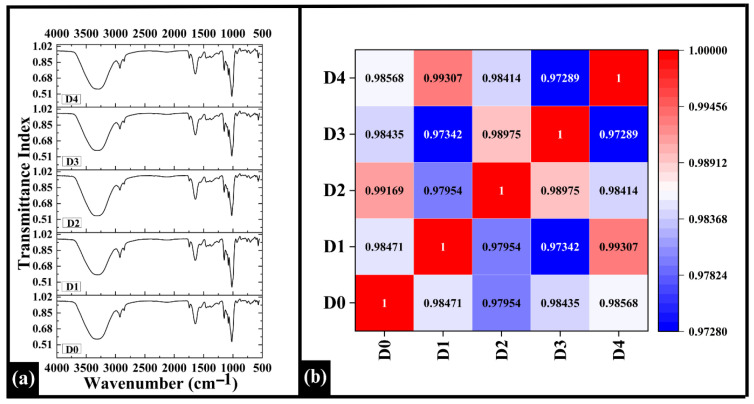
(**a**) Graph showing FTIR spectra of prepared WWB samples D0, D1, D2, and D4. (**b**) Heatmap of Spearman correlation coefficients of the FTIR profile. The data are presented as mean ± standard deviation. Different letters denote statistical differences among the samples (*p* < 0.05).

Fourier Transform Infrared (FTIR) spectroscopy identifies a sample’s molecular structure by measuring its absorption in the infrared region, where characteristic absorption bands for functional groups (like C-O, N-H, C-H, O-H, and C=O) reveal the presence of moisture, proteins, lipids, and carbohydrates [67,68]. On examining the FTIR spectra of the samples, the major peaks were observed at 3313 cm^−1^, 2926 cm^−1^, 1642 cm^−1^, 1150 cm^−1^, 1079 cm^−1^, and 1020 cm^−1^, as shown in Figure 9a. The broad absorption band around 3313 cm^−1^ might be attributed to O-H stretching vibrations, primarily due to water, starch, and protein (gluten), indicating the presence of moisture in the sample and hydrogen bonding interactions [69,70]. A peak at 2926 cm^−1^ corresponds to C-H stretching vibrations in the aliphatic CH2 and CH3 groups, which is correlated to lipid and fatty acid components present in WWF or rice bran oil [70]. A significant peak was observed at 1642 cm^−1^, attributed to the C=O stretching vibration from the amide I band, which represented the protein backbone, especially gluten and MR intermediates (e.g., Schiff bases and Amadori products) formed during baking [71]. The peaks at 1150 cm^−1^, 1079 cm^−1^, and 1020 cm^−1^ in the fingerprint region represent C-O stretching vibrations associated with carbohydrates such as starch, arabinoxylans, and cellulose associated with WWF [72].

The Spearman correlation coefficients among the FTIR spectra ranged from 0.97289 to 0.99307, demonstrating strong spectral similarity among all samples, as depicted in Figure 9b. The highest pairwise correlation was observed between D1 and D4 (ρ = 0.99307), while D3 and D4 exhibited the weakest relationship (ρ = 0.97289). Regarding the control (D0), D2 showed the highest correlation (ρ = 0.99169), indicating that its functional group composition remained most comparable to D0 despite the incorporation of DPG. This indicated that D2 has closer similarity in terms of functional group composition, suggesting minimal alteration in the carbohydrate and protein-associated absorption bands [73] with respect to D0.

#### 3.8.2. Peakwise Transmittance Index Analysis

**Figure 10:**
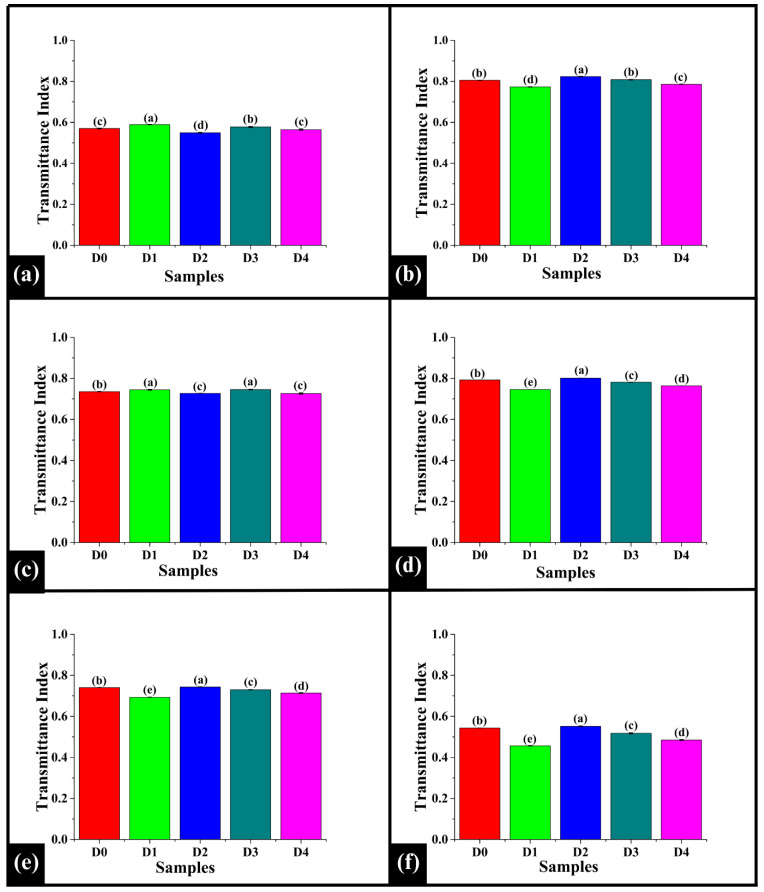
Bar graphs representing the transmittance index values of the WWB samples observed at (**a**) 3313 cm^−1^, (**b**) 2926 cm^−1^, (**c**) 1642 cm^−1^, (**d**) 1150 cm^−1^, (**e**) 1079 cm^−1^, and (**f**) 1020 cm^−1^ wavenumbers. The data are presented as mean ± standard deviation. Different letters denote statistical differences among the samples (*p* < 0.05).

The transmittance index was analyzed at the identified peaks 3313 cm^−1^, 2926 cm^−1^, 1642 cm^−1^, 1150 cm^−1^, 1079 cm^−1^, and 1020 cm^−1^. At 3313 cm^−1^, D0 exhibited a value of TI_3313_ of 0.570 ± 0.002, which was significantly lower than D1 (0.59 ± 0.00) and D3 (0.58 ± 0.00) (*p* < 0.05), while significantly higher than D2 (0.55 ± 0.00) (*p* < 0.05), as shown in Figure 10a. Among the DPG-added samples, D1 showed a significantly higher TI_3313_ value compared to D2, D3, and D4 (*p* < 0.05). D2 exhibited a significantly lower TI_3313_ value than D3 and D4 (*p* < 0.05), while D3 showed a significantly higher TI_3313_ value than D4 (*p* < 0.05). As per the observation, the increase in D1 and D3 values compared to D0 indicated the modification of the hydrogen bonding network due to DPG [74]. This might have influenced the interaction of water with starch and proteins [75]. The decreased value in D2 suggested increased absorption, which implied that the interaction of DPG with water, starch, and protein reorganized hydrogen bonds into a more stable network. Contrastingly, the statistical similarity of D4 compared to the control might be due to saturation of hydrogen bonding sites at high concentrations of DPG.

At 2926 cm^−1^, D0 showed a TI_2926_ value of 0.80 ± 0.00, significantly higher than D1 (0.77 ± 0.00) and D4 (0.79 ± 0.00) (*p* < 0.05), but statistically similar to D3 (0.81 ± 0.00) (*p* > 0.05), as illustrated in Figure 10b. The TI_2926_ value of D0 was significantly lower than D2 (0.823 ± 0.0015) (*p* < 0.05). In the DPG-added sample, D2 exhibited the highest TI_2926_ value, followed by D3, D4, and D1 in decreasing order (*p* < 0.05). The results exhibited a non-linear pattern for TI_2926_ values. In D1, a decrease in the TI_2926_ value was observed. This might result from the even lipid distribution, making the C-H bonds more exposed to infrared absorption [76]. The higher TI_2926_ value of D2 suggested a lower absorption, which could be attributed to the highly structured and stable matrix that might have shielded the lipid molecules, making the C-H bonds less exposed. Further, the subsequent decrease in TI_2926_ values in D3 and D4 was likely due to the saturation effect, where excess gum did not enhance matrix stability.

At 1642 cm^−1^, D0 displayed a TI1642 value of 0.735 ± 0.0005 (Figure 10c). The TI_1642_ value of D0 was significantly lower than D1 (0.74 ± 0.00) and D3 (0.74 ± 0.00) (*p* < 0.05), but significantly higher than D2 (0.73 ± 0.00) and D4 (0.73 ± 0.00) (*p* < 0.05). Among the DPG-added samples, the TI_1642_ value of D1 was significantly higher than D2 and D4 (*p* < 0.05), while statistically similar to D3 (*p* > 0.05). TI_1642_ values of D2 and D4 were observed to be statistically similar (*p* > 0.05). The increased TI1642 values of D1 and D3 indicated a more exposed protein backbone, causing a reduction in absorption at the Amide I band [77]. However, the decreased TI_1642_ value of D2 suggested the formation of a stable and rigid protein-starch matrix. Further, D4 also exhibited a lower TI_1642_ value, which was likely due to protein network collapse or aggregation due to excess DPG levels, indicating a saturation effect.

Further, at 1150 cm^−1^, the TI_1150_ value of D0 (0.79 ± 0.00) was significantly lower than D2 (0.80122 ± 0.0006) (*p* < 0.05) but significantly higher than D1 (0.74 ± 0.00), D3 (0.78 ± 0.00), and D4 (0.76 ± 0.00) (*p* < 0.05), as shown in Figure 10d. Among DPG-added samples TI_1150_ value of D1 was significantly lower than D2, D3, and D4 (*p* < 0.05). D3 showed a significantly higher TI_1150_ value than D4 (*p* < 0.05). D2 recorded the highest TI_1150_ value among all the samples (*p* < 0.05). A similar absorption pattern to 1150 cm^−1^ was observed at 1079 cm^−1^ and 1020 cm^−1^, as shown in Figure 10e and Figure 10f, respectively. These results provide a crucial understanding of the structural modifications of the carbohydrate matrix upon DPG addition. The band at 1150 cm^−1^ is a marker for the degree of starch crystallinity [78], while the bands at 1079 cm^−1^ and 1020 cm^−1^ reflect changes in the amorphous regions [79] and a hydrogen bonding network. This indicates a non-linear and concentration-dependent effect of DPG on the aforesaid interactions. In D1, the decreased TI values at these bands (1150 cm^−1^, 1079 cm^−1^, and 1020 cm^−1^) suggested a loss of crystalline order, signifying that the gum accelerated starch gelatinization [80]. In contrast, D2 exhibited the highest TI values, indicating a stronger water-binding capacity of DPG that might have helped to preserve the crystalline structure of starch. This result aligned with the improved crumb structure and moisture retention of D2, as observed in Section 3.2 and Section 3.3. However, at the higher DPG concentrations (D3 and D4), the TI values for all three bands decreased again, indicating that excessive DPG levels led to disruption of the starch granules [81].

In summary, D2 demonstrated a spectral pattern quite similar to D0, with minimum structural alteration, compared to the other DPG-containing samples. The reduced TI values at 3313 cm^−1^ and 1642 cm^−1^ in D2 indicate a stable and robust hydrogen bond network formed among the gum, water, and starch–protein complexes [69]. This is consistent with observations of microscopy, impedance, and deswelling, which support that D2 showed the most favorable and stable crumb structure.

### 3.9. Texture Profile Analysis

**Figure 11:**
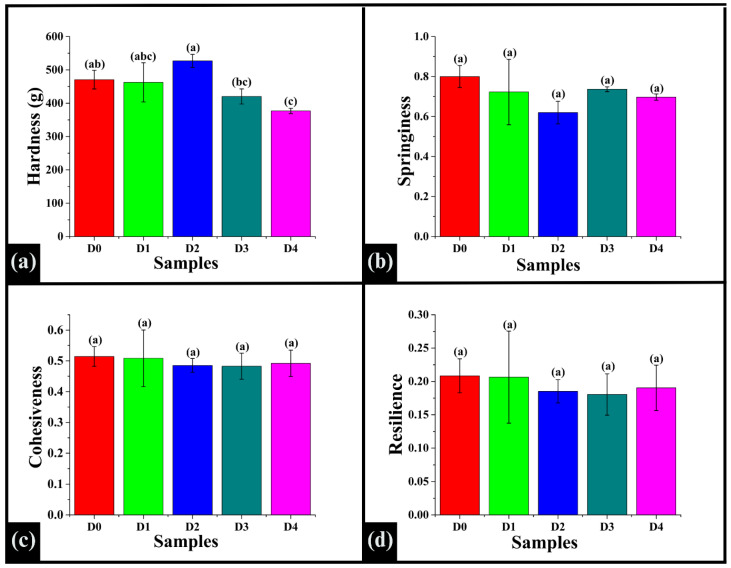
Bar graphs representing the textural parameters derived from the texture profile analysis (TPA) of the WWB sample: (**a**) hardness, (**b**) springiness, (**c**) cohesiveness, and (**d**) resilience. The data are presented as mean ± standard deviation. Different letters denote statistical differences among the samples (*p* < 0.05).

Texture profile analysis (TPA), also called the double compression test, was used to evaluate key textural parameters influencing sensory properties and consumer acceptability, such as hardness (the mechanical resistance to compression), springiness, cohesiveness, and resilience [82]. The WWB samples incorporated with various concentrations of DPG had a hardness value ranging between 376 g and 527 g. The hardness value of D0 (470.82 ± 28.08 g) was observed to be statistically similar to D1 (462.75 ± 58.79 g), D2 (527.00 ± 19.58 g), D3 (420.41 ± 22.60 g), and D4 (376.86 ± 8.13 g) (*p* > 0.05), as shown in Figure 11a. Among DPG-incorporated samples, the hardness value of D1 showed no significant difference compared to D2, D3, and D4 (*p* > 0.05). D2 exhibited a significantly higher hardness value compared to both D3 and D4 (*p* < 0.05). Further. the hardness values of D3 and D4 were statistically similar (*p* > 0.05). This showed that DPG incorporation influenced hardness in a non-linear pattern, where a moderate level of DPG enhanced firmness, and higher levels reduced hardness, which might be due to matrix softening and increased moisture retention. Springiness is a mechanical textural attribute that describes the ability of a food sample to return to its original form after being compressed [83,84]. The average springiness values ranged from 0.620 to 0.800, showing no significant difference among all the samples (*p* > 0.05), as depicted in Figure 11b. Cohesiveness measures the internal bonding strength of the food samples. It indicates how well the structure withstands a second deformation compared to its initial deformation [83]. In this study, the average cohesiveness values ranged from 0.48 to 0.51, in which all the samples exhibited statistical similarity (*p* > 0.05), indicating a negligible influence of DPG on bread crumbs (Figure 11c). Similarly, the resilience values of the samples, ranging from 0.180 to 0.208, were statistically similar (*p* > 0.05), as shown in Figure 11d. The significantly similar values of springiness, cohesiveness, and resilience across samples suggested that DPG did not affect these elastic properties, possibly due to the dominant role of the gluten-starch network [85]. This showed that DPG incorporation improved other aspects of bread quality without compromising the bread’s fundamental elastic texture, which is critical for consumer acceptance.

### 3.10. Stress Relaxation

**Figure 12:**
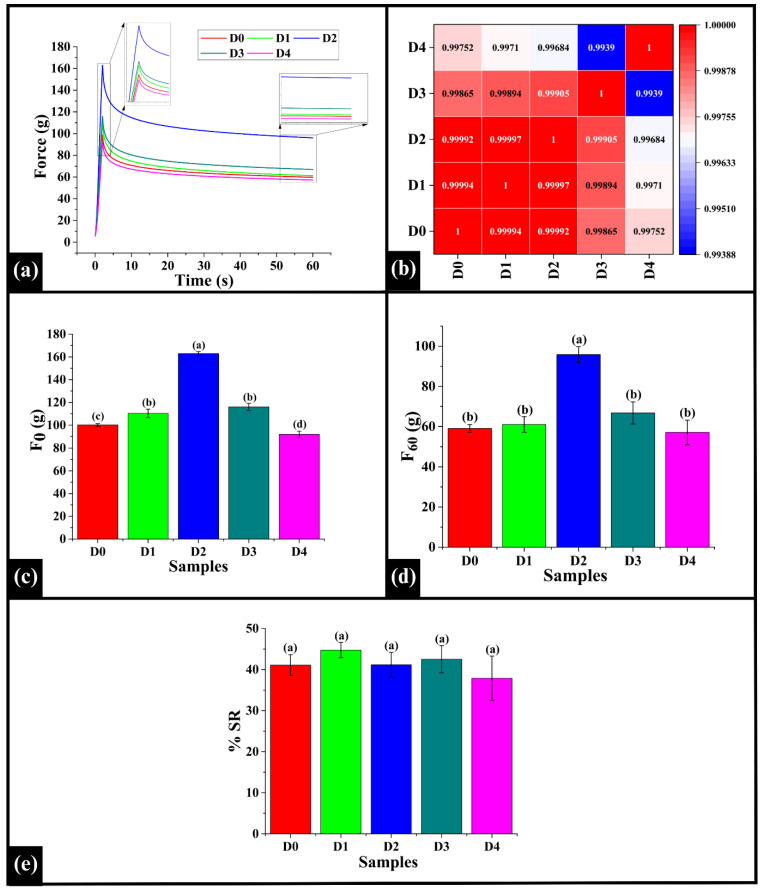
(**a**) Graphs representing stress relaxation (SR) profile of WWB samples, (**b**) Heatmap of Spearman correlation coefficients of SR profile, (**c**) F_0_ (**d**), and F_60_ (**e**) % SR of WWB samples. The data are presented as mean ± standard deviation. Different letters denote statistical differences among the samples (*p* < 0.05).

Stress relaxation (SR) in bread refers to the reduction in internal stress over time, during the deformation of the food sample, such as bread crumb or cake. This behavior arises due to the viscoelastic nature of the food matrix, which is influenced by components like gluten, starch, proteins, and moisture [86,87]. The stress relaxation graph (Figure 12a) illustrates the behavior of the bread crumb samples (D0–D4) for 60 s under constant deformation. In the initial phase (0–2 s), all samples exhibited a sharp rise in force, which reflects the elastic response of the crumb. After attaining the initial peak (F_0_), the force data for the relaxation phase (60 s) were recorded while the strain (deformation) was maintained [88]. All of the samples generally demonstrated a declining force over time that reached a residual force value (F_60_). Such a profile indicates the viscoelastic property of materials (herein, bread crumb) that possess both solid-like and fluid-like properties [89]. Among the samples, D2 showed the maximum initial force at the start of the experiment and maintained the highest residual force throughout the relaxation period, indicating a strong, firmer, and elastic texture. D0, D1, and D3 showed moderate stress relaxation behavior relative to other samples. On the contrary, D4 exhibited the most rapid stress decay, likely due to reduced internal mobility resulting from increased matrix compaction. Further, Spearman correlation analysis indicated a high level of positive correlation among all sample pairs (ρ = 0.9939−0.99997), suggesting that the viscoelastic response was similar across the samples, as shown in Figure 12b. The highest correlation was observed between D1 and D2 (ρ = 0.99997), indicating an identical viscoelastic mechanism among them. This can be associated with a consistent integration of DPG into the gluten-starch matrix [90,91] at these concentrations, with the higher DPG amount simply scaling the overall strength of the dough network in D2, without altering its fundamental SR behavior. In contrast, the lowest correlation coefficient was noted in D3 and D4 (ρ = 0.9939). Such an observation can be associated with the presence of DPG at a higher, supra-optimal concentration in D4. At this concentration, the DPG might be inducing structural interference either through agglomeration or competitive water binding, thereby altering the response of the bread matrix to the applied stress [91].

Subsequently, the SR parameters, specifically, maximum force (F_0_), residual force (force at the end of relaxation measurement, F_60_), and percentage stress relaxation (%SR), were analyzed. F_0_ signifies the maximum force required to initiate deformation, reflecting the firmness or elasticity of the crumb [15,16]. The F_0_ value of D0 was 100.26 ± 1.18 g, significantly lower than D1 (110.38 ± 3.70 g), D2 (162.95 ± 1.70 g), and D3 (116.06 ± 3.16 g) (*p* < 0.05), but significantly higher than D4 (91.83 ± 2.84 g) (*p* < 0.05), as shown in Figure 12c. Among the DPG-added samples, the F_0_ value of D1 was statistically similar to D3 (*p* > 0.05), while both were significantly lower than D2 (*p* < 0.05). Furthermore, F_60_ represents the residual elastic force that assesses the ability of the bread crumb to maintain its structure at the end of the experiment [90]. The F_60_ value of D0 (59.03 ± 1.92 g) was observed to be statistically similar to D1 (61.03 ± 3.91 g), D3 (66.77 ± 5.49 g), and D4 (57.11 ± 6.09 g) (*p* > 0.05), while D2 (95.81 ± 3.94 g) showed a significantly higher F_60_ value (*p* < 0.05) (Figure 12d). Among the DPG-added samples, D2 showed a significantly higher F_60_ value (*p* < 0.05). The results displayed that D2 had the highest F_0_ and F_60_ values, which indicated the formation of a crumb with increased firmness and a higher ability to maintain its structure under stress. This is consistent with the results of FTIR analysis, which confirmed the formation of stable hydrogen bonds between DPG and the components of dough [90].

Furthermore, the percent stress relaxation %SR, which quantifies the stress reduced over time under constant strain revealed no statistical differences in %SR values of D0 (41.10 ± 2.50%), D1 (44.75 ± 1.85%), D2 (41.18 ± 3.02%), D3 (42.53 ± 3.31%), and D4 (37.88 ± 5.42%) (*p* > 0.05), as depicted in Figure 12e. It can be observed that the %SR remained statistically similar across all the samples, suggesting that the addition of DPG did not substantially disrupt the balance between elastic and viscous behavior of the bread crumb due to the strengthening of the existing gluten-starch network [11]; however, the mechanics of the relaxation are not altered. In other words, the inclusion of DPG in the WWB samples improved the structural integrity of bread, but it did not negatively impact its viscoelastic behavior.

### 3.11. Assessment of Total Phenolic Content

**Figure 13:**
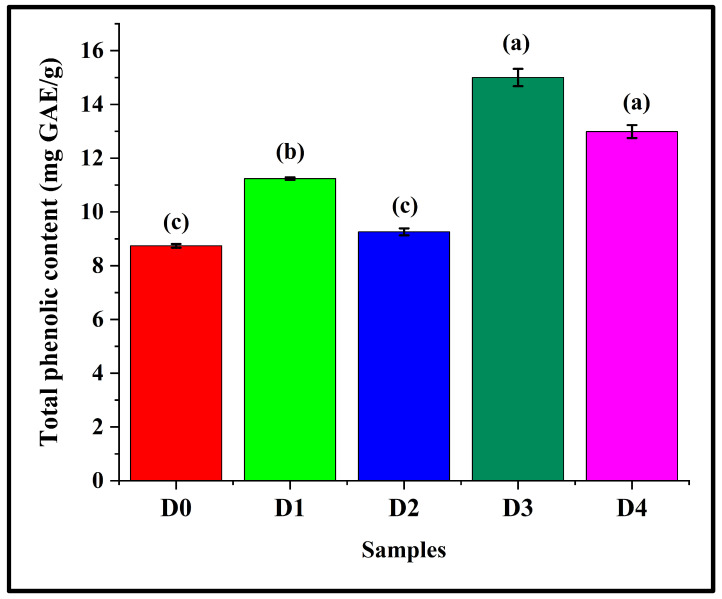
Graphs representing the total phenolic content of the bread samples expressed as mg GAE/g, where GAE stands for gallic acid equivalents. The data are presented as mean ± standard deviation. Different letters denote statistical differences among the samples (*p* < 0.05).

The TPEC of the bread samples was thoroughly evaluated. Figure 13 depicts the TPEC of the samples. It was observed that D0 had a TPEC value of 8.74 ± 0.07 mg GAE/g. When DPG was added, D1 exhibited a significant increase in the TPEC value (11.24 ± 0.05), as compared to D0 (*p* < 0.05). Further increase in the DPG content led to no marked alteration in the TPEC value of D2 (9.26 ± 0.13) than D0 (*p* > 0.05). D3 (15.00 ± 0.32) and D4 (12.99 ± 0.24) displayed a notable increment in the TPEC values when compared with D0 (*p* < 0.05). Among the formulations containing DPG, D2 showed the lowest TPEC value (*p* < 0.05). This might be due to the fact that the phenolic compounds in D2 would have been integrated into the hydrocolloid–starch–protein network, coupled with a structurally robust network as observed in stress relaxation analysis.

## 4. Conclusions

This study demonstrated the potential of DPG as a natural hydrocolloid to improve the physicochemical, functional, and textural properties of WWB. Samples containing varying levels of DPG (0 to 3% *w*/*w*) were prepared and assessed, of which D2 with 1% DPG exhibited the most desirable results. Microscopic evaluation of the crumb revealed that D2 showed a well-aerated crumb structure with evenly distributed medium-sized pores. This indicated enhanced fermentation activity and improved gas retention, forming an open crumb structure. It was observed that D2 exhibited better water retention (50.06 ± 0.41%) compared to other samples. Furthermore, the water activity of the samples did not significantly alter upon the addition of DPG. Moreover, the colorimetric analysis showed that increasing DPG concentration resulted in a decrease in L* values (46.69 ± 0.13). Additionally, D2 was observed to exhibit the highest initial (F0: 162.95 ± 1.70 g) and residual force (F60: 95.81 ± 3.94 g), suggesting the strengthening of the starch-gluten network. However, DPG did not impact the balance between the viscous and elastic components, as observed in the control sample. Finally, this investigation assessed the effect of DPG at a constant total hydration, and thus, the observed results reflect this specific condition. Future work should focus on two key areas for a complete characterization: (1) using a standardized dough consistency to isolate DPG’s intrinsic rheological effects and (2) quantifying its impact on the final nutritional profile (e.g., protein, fat, and fiber).

## Figures and Tables

**Table 1:** Composition of WWB samples at varying concentrations DTG: D0 (control, 0% *w*/*w* of DTG), D1 (0.5% *w*/*w* of DTG), D2 (1% *w*/*w* of DTG), D3 (2% *w*/*w* of DTG), and D4 (3% *w*/*w* of DTG).

Samples	Composition (g)						
	WWF	Water	DTG	Sugar	Salt	Yeast	Oil
D0	100.00	100.00	0.00	10.00	0.5	2.00	8.00
D1	100.00	99.50	0.50	10.00	0.5	2.00	8.00
D2	100.00	99.00	1.00	10.00	0.5	2.00	8.00
D3	100.00	98.00	2.00	10.00	0.5	2.00	8.00
D4	100.00	97.00	3.00	10.00	0.5	2.00	8.00

## Data Availability

The original contributions presented in the study are included in the article, further inquiries can be directed to the corresponding author.

## Abbreviations

WWB	Whole Wheat Bread
DTG	Date Tree Gum
TPA	Texture Profile Analysis
SR	Stress Relaxation
FTIR	Fourier Transform Infrared Spectroscopy
EIS	Electrical Impedance Spectroscopy
